# Heterologous Expression of the Core Genes in the Complex Fusarubin Gene Cluster of *Fusarium Solani*

**DOI:** 10.3390/ijms21207601

**Published:** 2020-10-14

**Authors:** Tobias Bruun Pedersen, Mikkel Rank Nielsen, Sebastian Birkedal Kristensen, Eva Mie Lang Spedtsberg, Wafaa Yasmine, Rikke Matthiesen, Samba Evelyne Kabemba Kaniki, Trine Sørensen, Celine Petersen, Jens Muff, Teis Esben Sondergaard, Kåre Lehmann Nielsen, Reinhard Wimmer, Jens Laurids Sørensen

**Affiliations:** 1 Aalborg University Esbjerg, Department of Chemistry and Bioscience, Niels Bohrs Vej 8, 6700 Esbjerg, Denmark; tbp@bio.aau.dk (T.B.P.); mrn@bio.aau.dk (M.R.N.); sbk@bio.aau.dk (S.B.K.); espedt16@student.aau.dk (E.M.L.S.); wyasmi14@student.aau.dk (W.Y.); rmatth14@student.aau.dk (R.M.); skabem15@student.aau.dk (S.E.K.K.); jm@bio.aau.dk (J.M.); 2 Aalborg University Aalborg, Department of Chemistry and Bioscience, Fredrik Bajers Vej 7H, 9220 Aalborg, Denmark; trso@bio.aau.dk (T.S.); cepe@bio.aau.dk (C.P.); tes@bio.aau.dk (T.E.S.); kln@bio.aau.dk (K.L.N.); rw@bio.aau.dk (R.W.)

**Keywords:** polyketides, yeast, heterologous expression, Fusarium, pigments, fungi, bostrycoidin

## Abstract

Through stepwise recreation of the biosynthetic gene cluster containing *PKS3* from *Fusarium solani*, it was possible to produce the core scaffold compound of bostrycoidin, a red aza-anthraquinone pigment in *Saccharomyces cerevisiae*. This was achieved through sequential transformation associated recombination (TAR) cloning of *FvPPT*, *fsr1*, *fsr2*, and *fsr3* into the pESC-vector system, utilizing the inducible bidirectional galactose promoter for heterologous expression in *S. cerevisiae*. The production of the core metabolite bostrycoidin was investigated through triplicate growth cultures for 1–4 days, where the maximum titer of bostrycoidin was achieved after 2 days of induction, yielding 2.2 mg/L.

## 1. Introduction

Within the realm of natural product chemistry, a specific group of compounds known as polyketides has been vigorously studied for many years because of their structural diversity, bioactivity, and industrial applicability. A few examples such as the cholesterol lowering compound lovastatin, anti-cancer compound cytochalasin E, and the antibiotic tetracyclines display only a fraction of the diverse beneficial properties of polyketides [1,2,3]. Other properties often associated with polyketides are anti-bacterial and anti-fungal activity, pigmentation, and possibly even electrolytic capacity in batteries [4]. In nature, these compounds are a result of the complex secondary metabolism of plants, fungi, and bacteria, expressing not only polyketides, but a plethora of other bioactive molecules. This renders purification of a single compound from natural sources laborious and difficult because crude extracts comprise a cocktail of metabolites.

In order to obtain high-titers of purified polyketides, a popular strategy is to engineer heterologous expression hosts that display a minimalistic secondary metabolite profile, while being well suited for large-scale fermentation [5]. An example of such a host is *Saccharomyces cerevisiae*, with a highly developed molecular toolbox [6], a simplistic metabolism [7], and a generally regarded as safe (GRAS) status organism suited for fermentation [8]. Model polyketides 6-methylsalisylic acid (6-MSA) and triacetic acid lactone (TAL) have been heterologously expressed in *S. cerevisiae* with titers of 1.6–1.7 g/L, which further emphasizes the potential for production of polyketides in this host [9,10]. In order to obtain these levels of production, the primary metabolism of *S. cerevisiae* was optimized for higher flux towards the acetyl- and malonyl-Coenzyme A (CoA) pathways, rendering a higher concentration of precursor molecules for polyketide assembly. More complex polyketides of multiple ring-structures such as the pigment compounds bikaverin and rubrofusarin from the *Fusarium* species complex have also been expressed in *S. cerevisiae*, although lower titers of production were obtained, 3.65 mg/L and 1.1 mg/L, respectively, owing to the structural complexity of these polyketides [11,12].

In this study, we target the core genes of the fusarubin biosynthetic gene cluster in *F. solani*. The gene cluster is found in all genome sequenced Fusaria [13] and has previously been assigned to the production of 8-*O*-methylfusarubin in *F. fujikuroi* [14] and the aza-anthraquinone bostrycoidin in *F. graminearum* [15]. Besides the polyketide synthase (PKS) FSR1 (PKS3, PGL1), two enzymes were needed to yield the end products in these species—the *O-methyltransferase* FSR2 and the FAD-binding monooxygenase FSR3. For effective heterologous expression of polyketides in *S. cerevisiae*, it is necessary to co-express a 4’-phosphopantetheinyl transferase (PPTase) along with the tailoring enzymes as the acyl carrier protein (ACP)-domain of the polyketide synthase requires additional phosphopantetheinylation to drive the iterative enzymatic process of elongating the ketide-backbone. Therefore, a PPTase native to *F. verticillioides* was also introduced into the expression strains.

**Figure 1:**
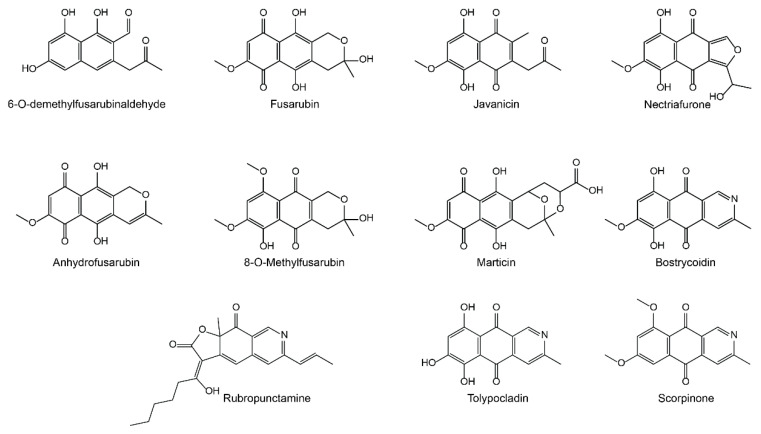
Structures of prominent fusarubins isolated from members of the *Fusarium solani* species complex as well as the structurally similar rubropunctamine from Monascus spp. [18], tolypocladin from *Tolypocladium inflatum* [19], and scorpinone from *Amorosia littoralis* [20].

*PKS3* has previously been heterologously expressed in *Escherichia coli*, which resulted in production of 6-*O*-demethylfusarubinaldehyde [16]. This compound is also predicted to be the entry molecule in the biosynthetic pathways in *F. fujikuroi* and *F. graminearum.* The production of fusarubins appears to be more enigmatic in the *F. solani* species complex, where more than 50 related compounds have been isolated, with fusarubin, javanicin, and bostrycoidin as the most prominent [17] (Figure 1).

**Figure 2:**
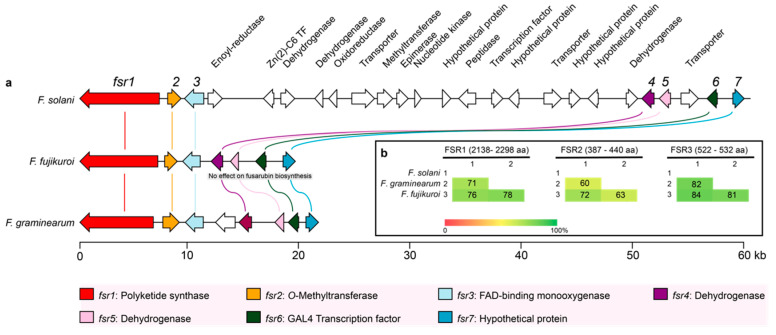
(**a**) Overview of the fusarubin gene clusters in *F. solani*, *F. fujikuroi*, and *F. graminearum*, where the seven conserved genes have been highlighted. The additional genes were tentatively identified using protein prediction software InterPro and NCBI’s Conserved Domain Database (CDD) [22,23]. (**b**) Similarity matrices (% identity) of the amino acid sequences of the three core enzymes based on clustalW alignments.

Furthermore, the PKS3 gene cluster of *Fusarium solani* sp. exhibits a significant diversity from other members of the *Fusarium* genus [21], as it comprises the seven core genes (*fsr1–7*) with 17 additional genes, including some genes with potential tailoring capacity, which could explain the higher diversity of fusarubins produced by *Fusarium solani* (Figure 2a). In this study, transcription of the genes was controlled by the inducible *Gal1/10* promotor, leveling the transcription rate dissimilar to natural flux in wild-type transcription.

The aim of study was to determine whether the three core genes contribute to the metabolic diversification through heterologous expression in *S. cerevisiae*.

## 2. Results and Discussion

### 2.1. Comparison of the PKS3 Gene Clusters

In *F. fujikuroi*, the end product in the pathway is predicted to be 8-*O*-methylfusarubin, which has an additional methyl group compared with fusarubin produced by *F. solani* as one of the main products. As this difference is most likely mediated by FSR2, we performed an alignment analysis of the three core enzymes (Figure 2b). The alignment showed that the FSR3 is the most conserved among the three analyzed enzymes in *F. solani*, *F. fujikuroi*, and *F. graminearum*, with sequence identity ranging from 81 to 84% on amino acid level. FSR2 on the other hand displayed the largest variation, where *F. graminearum* displayed only 60 and 63% sequence identity to the orthologues in *F. solani* and *F. fujikuroi*, respectively.

### 2.2. Heterologous Expression of the Three Fusarubin Core Genes

**Figure 3:**
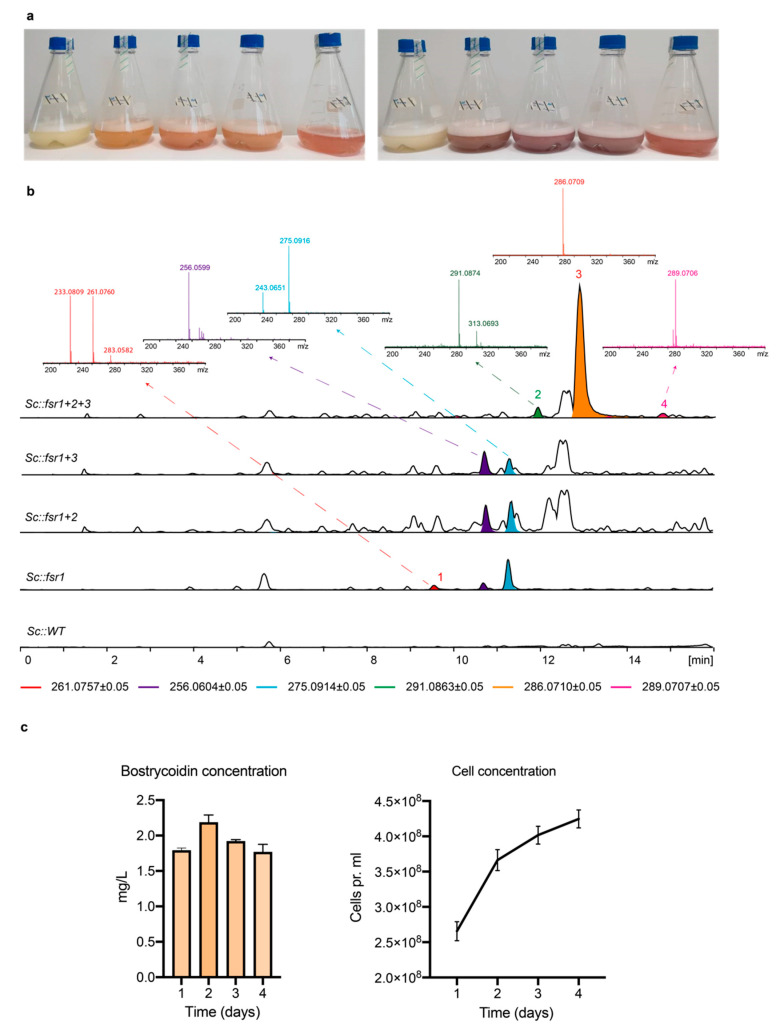
(**a**) Growth cultures after 24 h and 48 h, containing, from left to right, Sc::WT, Sc::*fsr1*, Sc::*fsr1+2*, Sc::*fsr1+3,* and Sc::*fsr1+2+3*. (**b**) Total ion chromatograms (TIC, black) and extracted ion chromatogram (EIC; colored, intervals listed below) of the yeast cultures. The selected mass spectra of compounds mentioned in the text have been included and the following compounds are indicated: (1) 6-O-demethylfusarubinaldehyde, (2) javanicin, (3) bostrycoidin, and (4) anhydrofusarubin. (**c**) Results from triplicate growth cultures grown for 1–4 days, showing the production of bostrycoidin alongside the cell concentration.

In order to determine the contributions of the three core genes to the biosynthetic pathway, we generated the four combinations (*S. cerevisiae* BY4743::*FvPPT+fsr1*), (*S. cerevisiae* BY4743::*FvPPT+fsr1+2*), (*S. cerevisiae* BY4743::*FvPPT+fsr1+3*), and (*S. cerevisiae* BY4743::*FvPPT+fsr1+2+3*), from here on referred to as Sc::*fsr1*, Sc::*fsr1+2*, Sc::*fsr1+3,* and Sc::*fsr1+2+3*, respectively. For each combination, a representative transformant was grown in conditions inducing the *Gal1/10* promotor system for two days, alongside a wild type *S. cerevisiae*. Changes in color were visible after one day (left) and evident already after two (right) (Figure 3a).

The subsequent metabolite analyses showed that three new peaks emerged with *fsr1* as the sole member of the gene cluster, when compared with the spectrum of Sc::WT. One of these peaks matched the expected spectrum for 6-*O*-demethylfusarubinaldehyde (C_14_H_12_O_5_, [M+H]^+^_exp_: 261.0757; [M+H]^+^_obs_: 261.0760), which is the expected release product of FSR1. Another compound identified in Sc::*fsr1* was tentatively assigned to C_15_H_14_O_5_ ([M+H]^+^_exp_: 275.0914; [M+H]^+^_obs_: 275.0916). So far, attempts to isolate and identify this compound have not been successful in the present or previous studies [24]. The hypothetical next step towards bostrycoidin is a spontaneous ammonium incorporation, resulting in 6-*O*-demethyl-5-deoxybostrycoidin anthrone (C_14_H_11_N_1_O_3_; [M+H]^+^_exp_: 256.0604) [15,25]. However, this compound was not detected in the extracts of any of the strains. Instead, another peak was observed in Sc::*fsr1* with a suggested chemical formula of C_14_H_9_N_1_O_4_ ([M+H]^+^_exp_: 256.0604; [M+H]^+^_obs_: 256.0599), which could be an oxidized form of 6-*O*-demethyl-5-deoxybostrycoidin anthrone. The latter two tentatively assigned compounds were also observed when *fsr1* was expressed with either *fsr2* or *fsr3*. However, we were not able to determine other pathway intermediates.

Co-expression of *fsr1–3* resulted in the production of javanicin, anhydrofusarubin, and bostrycoidin, with the latter as the dominating peak. The highest titer of bostrycoidin was achieved after 2 days of induction, reaching 2.2 mg/L (Figure 3c), very similar in titer level when compared with other complex polyketide expression studies in *S. cerevisiae* [11,12]. An explanation for the slight decrease in bostrycoidin concentration after 3 and 4 days could be the accumulation of bostrycoidin within the cell pellet due to limited solubility of bostrycoidin in the media. It was evident that the cell pellet of the cultures from day 3 and 4 was significantly larger and more dark red than that of cultures from day 1 and 2, indicating an intercellular accumulation (Figure S1). The observation of 6-*O*-demethylfusarubinaldehyde in Sc::*fsr1* confirms its identity as the PKS release product [14,15]. The following biosynthetic pathway can then branch out in two main directions, ending in bostrycoidin on one side and javanicin and anhydrofusarubin on the other side. The ability of Sc::*fsr1+2+3* to produce bostrycoidin suggests that ammonia is incorporated by a conserved fungal enzyme or, more likely, non-enzymatically as proposed previously [15,25]. Given the structural similarities of tolypocladin [19], scorpinone [20], and bostrycoidin, it seems very likely that the compounds share similar pathways including the amination step. A similar situation has also been observed in *Monascus* spp., where spontaneous amination with NH_3_ is observed in the pathways, leading to the red pigment rubropunctamine and monascorubramine [18].

**Figure 4:**
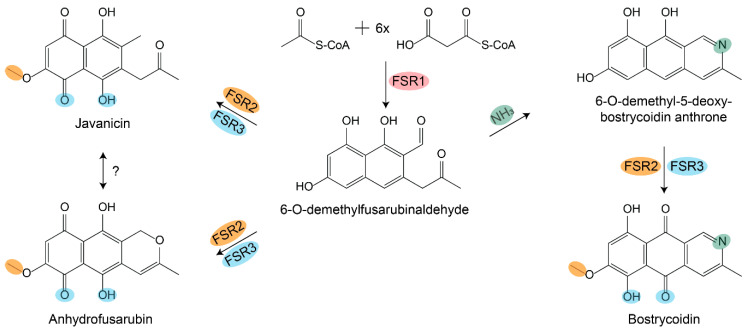
Suggested biosynthetic pathways leading to bostrycoidin, javanicin, and anhydrofusarubin. The polyketide synthase FSR1 is responsible for the first step, which results in 6-*O*-demethylfusarubinaldehyde. Non-enzymatic incorporation of ammonia followed by oxygenation and o-methylation by FSR3 and FSR2 result in bostrycoidin. The two enzymes can also act independently on ammonia incorporation, which results in anhydrofusarubin and javanicin.

Anhydrofusarubin is structurally very similar to bostrycoidin, differing in the presence of an oxygen or nitrogen molecule in the C ring. It thus seems likely that anhydrofusarubin biosynthesis follows the path of bostrycoidin, where formation of the third ring occurs in one of the early steps (Figure 4). Whether javanicin is produced directly by FSR2 and FSR3 en route to anhydrofusarubin remains unknown. Alternatively, it could result from reopening of ring C in anhydrofusarubin. The ability to catalyze oxygenation and methylation reactions towards anhydrofusarubin and bostrycoidin suggests that the two enzymes have a promiscuous nature. It is striking that FSR2 from *F. solani* only adds one methyl group, while the orthologue from *F. fujikuroi* performs two. The alignments showed that the two enzymes are relatively similar (Figure 2b), but more complex comparison studies of their tertiary structures may shed light on the nature of the catalytic differences.

In our study, we only observed a minor portion of the fusarubins that have been reported in literature. This leaves the possibility that the remaining compounds are produced spontaneously or through actions of the enzymes encoded by the additional tailoring genes in the cluster from *F. solani*. Furthermore, the promiscuous nature of a 4´-phosphopantetheinyl transferase (PPTase) from *Fusarium verticillioides* exhibited adequate function for regenerating the acyl carrier protein (ACP) domain of the polyketide synthase (*fsr1*) from the closely related *Fusarium solani.*

## 3. Materials and Methods

### 3.1. Strains

Yeast cloning and heterologous expression were performed utilizing *S. cerevisiae* BY4743 (*genotype:* MATα, his3Δ1, leu2Δ0, lys2Δ0, met15Δ0, ura3Δ0) ATCC 201390 and electroporation was performed on bacterial strain *Escherichia coli* DH5α. The genome sequenced *F. solani* FGSC9596 [26] was used as a donor of DNA for amplification of *fsr2*.

### 3.2. Pairwise Alignment

Pairwise comparison of the protein encoded by the cluster genes was performed in CLC Main Workbench 8.1 using the clustalW algorithm (CLC Bio-Qiagen, Aarhus, Denmark) as previously described [27]. The sequences of FSR1-3 from *F. fujikuroi* (FFUJ_03984-86), *F. graminearum* (FGSG_09182-84), and *F. solani* (NECHADRAFT_101778-80) were retrieved from GenBank.

### 3.3. Enzymes, Oligonucleotides, and Plasmids

All enzymes were purchased from Thermo Fisher Scientific (Waltham, MA, USA) and oligonucleotides were designed using Primer3Plus software [28] and purchased from Eurofins Genomics (Ebersberg, Germany). The primer table including sequences can be found in Table S1. An overview of purchased and constructed plasmids can be found in Table S2 along with restriction enzymes used for digestion.

### 3.4. Gene Isolation and Synthesis

Coding sequences for *fsr1* and *fsr3* were codon optimized towards expression in yeast and synthesized (GenScript), while *fsr2* was amplified from genomic DNA prepared through DNeasy Plant Mini Kit (Qiagen, Hilden, Germany) from *F. solani* and the two exons were fused together to obtain intron-free CDS of *fsr2* using transformation associated recombination (TAR)-cloning. To facilitate polyketide formation in *S. cerevisiae*, we co-expressed *fsr1* with the *Sfp*-type phosphopantetheinyl transferase from *F. verticillioides* (FVEG_01894).

The four target genes were amplified by PCR using the primers, which contained 25–30 bp identical sequences to the target insert site of the vector expression system of pESC-URA or pESC-LEU, used for auxotrophic selection on uracil (URA) and leucin (LEU) deficient media. The PCR reactions were performed in a 150 μL volume containing (30 μL High Fidelity buffer, 0.2 mM dNTP-mix, 0.2 mM forward/reverse Primer, 0.5 units Phusion HSII polymerase (New England Biolabs, Ipswich, MA, USA), 97.5 μL MQ H_2_O, and 1 ng template DNA), split into 3 × 50 μL PCR-tubes.

### 3.5. Cloning

Prior to assembly of the expression plasmid, the receiving pESC-URA or pESC-LEU were linearized by double restriction enzyme digest (2 μL Fast Digest Buffer (Thermo Fisher Scientific, Waltham, MA, USA), 2 μL plasmid miniprep (QIAquick Miniprep kit), 1 μL FD Enzyme 1 (Thermo Fisher Scientific, Waltham, Massachusetts, USA), 1 μL FD Enzyme 2 (Thermo Fisher Scientific, Waltham, Massachusetts, USA), and 14 μL MQ H_2_O at 37 °C for 15 min). The digested plasmids were separated on 1% w/v agarose gel and subsequently purified with the QIAquick Gel Extraction Kit (Qiagen, Hilden, Germany). TAR-cloning was performed according to the LiAc/PEG3350 protocol [29]. Selection of *S. cerevisiae* transformants was conducted using synthetic complete (SC) agar plates, lacking either uracil, leucine, or both [24].

**Figure 5:**
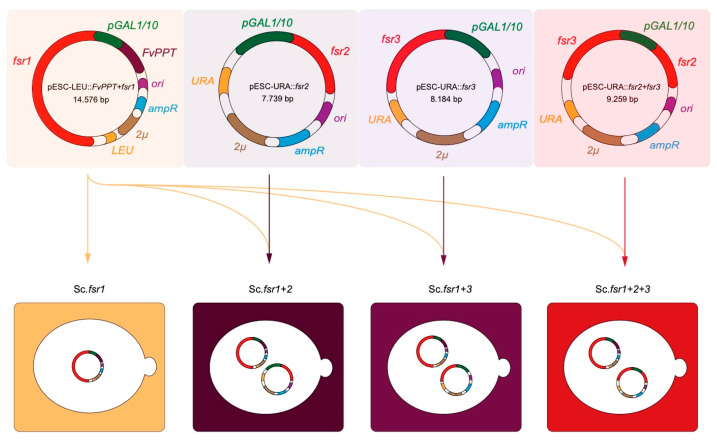
Final constructs of expression vectors utilizing the URA or LEU auxotrophic selection in *S. cerevisiae* BY4743, GAL1/10 inducible promotor, bacterial (*ori*), yeast (*2μ*) origin of replication along with the bacterial selection marker (*amp*^R^). Sc::*fsr1* contains only the *fsr1*-gene, the main biosynthetic gene; a type I non-reducing PKS (NR-PKS) and *FvPPT*, a 4´-phosphopantetheinyltransferase (PPTase) from *Fusarium verticillioides* for successful phosphopantetheinylation of the acyl carrier protein (ACP)-domain of *fsr1*. The following strains contain a dual vector system. Sc::*fsr1+2* contains both *FvPPT, fsr1,* and the *fsr2*-gene, a O-methyltransferase. Sc::*fsr1+3* contains *FvPPT, fsr1,* and the *fsr3-*gene encoding a monooxygenase. Finally, Sc::*fsr1+2+3* contains all core biosynthetic genes involved in the synthesis and tailoring, *FvPPT*, *fsr1, fsr2,* and *fsr3*.

The plasmid from the obtained transformants were recovered through adaptation of the QIAprep Spin Miniprep Kit (Qiagen, Hilden, Germany) protocol, adding 5 μL Zymolyase-mix (2 mg/mL Zymolyase in 0.5 M sorbitol) (MP Biomedicals Germany) to the P1 Buffer step and incubating at 37 °C for 1 h. The purified plasmids were electroporated into *E. coli* for proliferation and subsequently initially validated using Sanger-sequencing (Eurofins, Ebersberg) using the primers listed in Table S1 before full plasmid sequencing was performed using a MinION Flow Cell for nanopore sequencing (See 3.8 Plasmid verification). The verified plasmids were transformed into *S. cerevisiae* to obtain combinations where *fsr1* was expressed alone (Sc::*fsr1*), together with either *fsr2* (Sc::*fsr1+2*) or *fsr3* (Sc::*fsr1+3*), and all together (Sc::*fsr1+2+3*), as seen in Figure 5.

### 3.6. Production of Fusarubins Using Galactose Induction

Single colonies of transformants carrying biosynthetic genes were inoculated in 10 mL appropriate selective drop-out medium containing 2% glucose overnight at 30 °C, 200 rpm. Overnight cultures were vortexed and cell titers were estimated using a NanoDrop 2000 c (Thermo Fisher Scientific, Waltham, MA, USA). The cells were transferred into 1000 mL baffled shake flasks containing 250 mL selective media with 2% raffinose (D(+)-raffinose pentahydrate, Acros organics, China) to an OD600 = 0.2. The cells were grown for approximately 9–10 h at 30 °C, 200 rpm until an OD600 at 1 was achieved. The cultures were spiked with 25 mL 40% galactose (D(+)-galactose, VWR chemicals, Belgium) and maintained at 30 °C and 200 rpm for 1–4 days to induce expression of the fungal biosynthetic genes.

### 3.7. Chemical Analysis

After cultivation, the cells were pelleted by centrifugation at 5.000 G for five minutes. Approximately 300 mL of media was decanted into a 1000 mL bluecap flask and extracted as previously described for aurofusarin in *F. graminearum* [30]. The dried extracts were dissolved in 2 mL methanol and analyzed on a Hitachi Elite LaChrom HPLC in accordance with the chemical analysis performed in [24]. The identities of javanicin, anhydrofusarubin, and bostrycoidin were determined using data from previous experiments where they were isolated and structurally verified in *F. solani* [24].

### 3.8. Plasmid Verification

Library preparation was performed using the Rapid Barcoding Kit SQK-RBK004 (Oxford Nanopore Technologies, Oxford, UK) according to the manufacturer’s guidelines and subsequently sequenced using a R.9.4.1 (FLO-MIN106D) MinION Flow Cell (Oxford Nanopore Technologies, Oxford, UK; Figure S2). The flow cell had previously been used, but was washed according to the manufacturer’s instructions with the Flow Cell Wash Kit EXP-WSH003 (Oxford Nanopore Technologies, Oxford, UK). Fast5 files were basecalled and demultiplexed using GPU driven Guppy v3.6.1 (Oxford Nanopore Technologies, Oxford, UK) with the following model: dna_r9.4.1_450bps_hac.cfg. Filtlong v0.2.0 (https://github.com/rrwick/Filtlong) was used to filtrate the reads to a minimum length of 100 and minimum quality of 80. Minimap2 v2.17 [31] was used to create overlaps between the reads and the assemblies were generated by Miniasm v0.3 [32]. The assemblies were first polished with Racon v1.3.3 [33] and then with two rounds of Medaka v1.0.1 (https://github.com/nanoporetech/medaka); both with default settings.

## Supplementary Materials

Supplementary materials can be found at https://www.mdpi.com/1422-0067/21/20/7601/s1.
