# Supplementary material for: Heterologous Expression of the Core Genes in the Complex Fusarubin Gene Cluster of Fusarium Solani
Source: Int J Mol Sci. 2020 Oct 14;21(20):7601. doi: 10.3390/ijms21207601 (PMC7589453; doi:10.3390/ijms21207601)
Supplement: Supplementary file 1 [file ijms-21-07601-s001.pdf]

Supplementary material

Table S1. This table contains the primer sequences of both the primers used for gene-amplification and the primer used for initial sanger-sequencing in fragments of around 700 bp, containing at least 50 bp overlap between each fragment.

Gene	Fragments	Product size (bp)	Name	TAR-region and annealing sequence for PCR
Fsr1	2	3274	Fsr1.1-fw	AAA ATT CGA ATT CAA CCC TCA CTA AAG GGC ATG ACA GAC AAC TTA AAA TTA TAC TTA TTC G
			Fsr1.1-rv	CCT TCA AAG CTG CAC ACA AA
		3291	Fsr1.2-fw	TTC CAT ACG CAT TCC ATT CA
			Fsr1.2-rv	ACA ACC TTG ATT GGA GAC TTG ACC AAA CCT TCA AAC TCT TGG ACC CCA CA
Fsr2	2	563	Fsr2.1-fw	ATA CTT TAA CGT CAA GGA GAA AAA ACC CCG ATG CAC AAG ACT GAA AGA GAC G
			Fsr2.1-rv	GGT GGC GGT AGA ACC GCT GCT TCC ACC AAC ATC AAC GAC CTT GGC CTC T
		618	Fsr2.2-fw	AAG GCT CTG GGA GAG GCC AAG GTC GTT GAT GTT GGT GGA AGC AGC GGT
			Fsr2.2-rv	GAT CTT AGC TAG CCG CGG TAC CAA GCT TAC CTA AGC ATG CCC ATT CAG ACC
Fsr3	1	1599	Fsr3-fw	AAA ATT CGA ATT CAA CCC TCA CTA AAG GGC ATG CAA ATC AAC GAC CAA AC
			Fsr3-rv	GCC GAC AAC CTT GAT TGG AGA CTT GAC CAA CTA TGC CCA GTC ACC GTC TT
FvPPT	1	879	FvPPT-fw	GTT GAT TTC CGA AGA AGA CCA TGT CCT CAG CAC AAT CAT CA
			FvPPT-rv	GCT AGC CGC GGT ACC AAG CTT TAT GAT TTA GGA GCC TTT TCA CC

Sequencing primers

Fsr1	10	+700	Fsr1.1	ATGTATATGGTGGTAATGCCATG
		+700	Fsr1.2	AATTTGCCATTGACTTCTAGACC
		+700	Fsr1.3	TACATCAGCTACTCCATTTGG
		+700	Fsr1.4	AGCAGCTGTTGATCCATATAC
		+700	Fsr1.5	TACTTTTACTGGTCAAGGTGC
		+700	Fsr1.6	GGCTAACCATTCTAGACAATCAG
		+700	Fsr1.7	GGGCATCTCAATCAGCAAC
		+700	Fsr1.8	GCAAATGGTTCATCTGGTATCC
		+700	Fsr1.9	TTAACAACGCTCATTCTGGTAC
		+700	Fsr1.10	CAGAGCCATGGTTGTTAGATTG
Fsr2	2	+700	Fsr2.1	CAACATTTTCGGTTTGTATTACTTC
		+700	Fsr2.2	GCCAAGGTCGTTGATGTTG
Fsr3	3	+700	Fsr3.1	ATGTATATGGTGGTAATGCCATG
		+700	Fsr3.2	AAGTTGTCGAATACTGGGAAG
		+700	Fsr3.3	GATACCAAAGAGTTTCATGTGGTC
FvPPT	2	+700	FvPPT.1	CAACATTTTCGGTTTGTATTACTTC
		+700	FvPPT.2	ATGCGTACACGCGTCTGTACAG

TableS 2. This table contains the different plasmids utilized in the project, both the native plasmids used as expression vectors, but also plasmids purchased containing the synthetically derived codon optimized genes.

Plasmid	Gene inserted	Purchased	Constructed	Restriction enzymes for linearization	Resulting plasmid
pESC-URA	*empty*	•			
pESC-LEU	*empty*	•			
pUC57	*fsr1*	•			
pUC57	*fsr3*	•			
pUC57	*FvPPT*	•			
pESC-LEU	*FvPPT*		•	XhoI/HindIII	pESC-LEU::*FvPPT*
pESC-LEU+*FvPPT*	*fsr1*		•	NotI/BglII	pESC-LEU::*FvPPT+fsr1*
pESC-URA	*fsr2*		•	BamHI/XhoI	pESC-URA::*fsr2*
pESC-URA	*fsr3*		•	NotI/BglII	pESC-URA::*fsr2*
pESC-URA+*fsr3*	*fsr2*		•	NotI/BglII	pESC-URA::*fsr2+3*

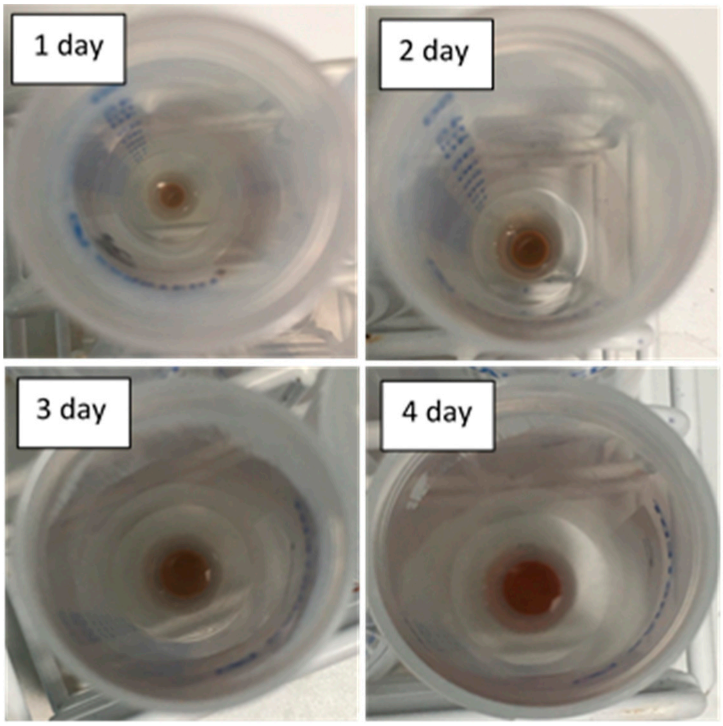

Figure S1. Accumulation of pigments in cells of Sc::*fsr1+2+3*. The cells were collected from 50 mL medium and pelleted by centrifugation 5.000G for five minutes.

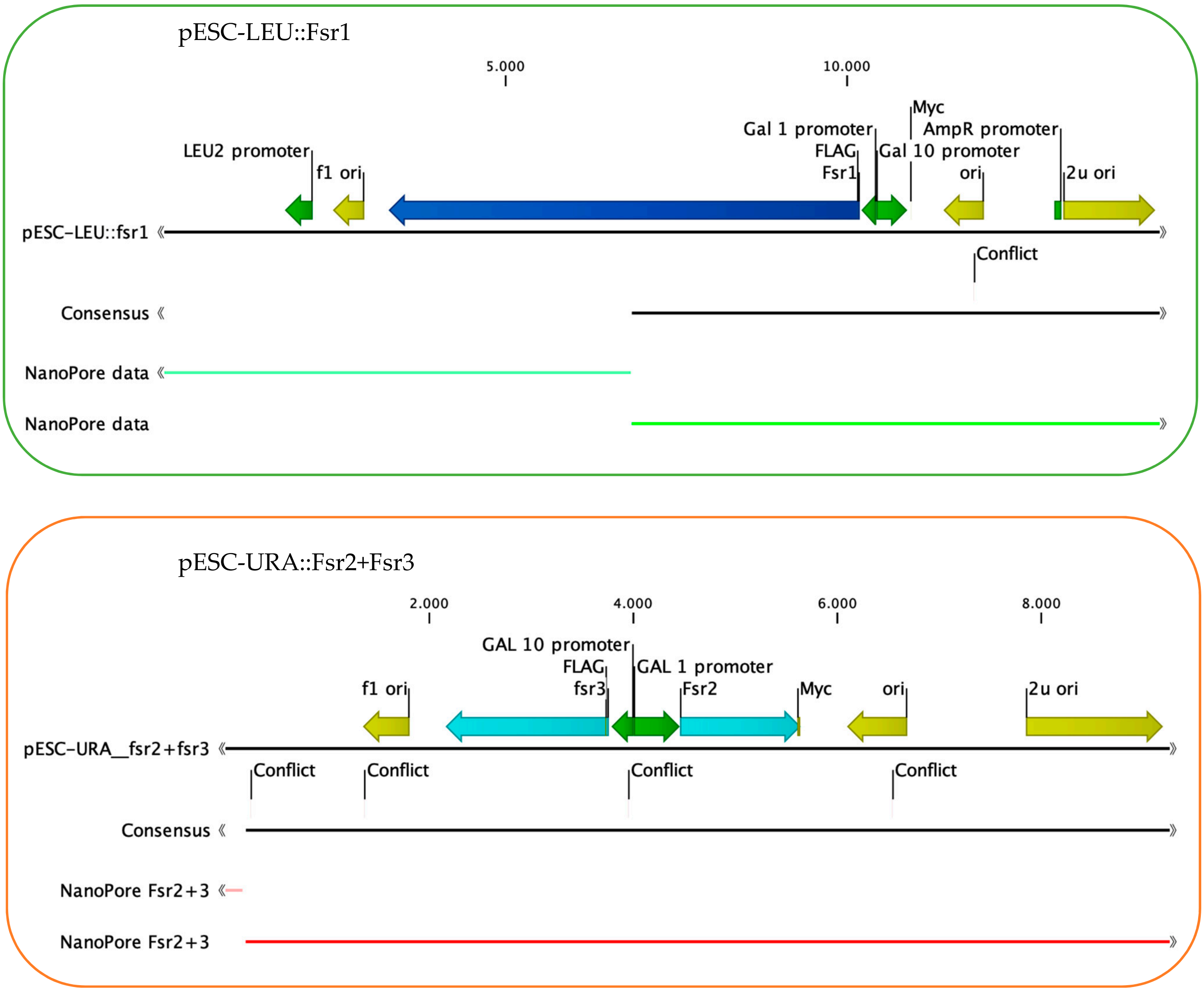

Figure S2. Alignment of NanoPore sequencing data to reference plasmids created in CLC Main Workbench 8, illustrating only a few conflicting incidents which are negligible and none of them occur within the introduced genes.
